# Aligning outcomes: DLBCL prognosis at a 4th Level University Hospital in Bogotá is comparable to high-income nations, identification of additional prognostic markers for overall survival and relapse

**DOI:** 10.3332/ecancer.2024.1717

**Published:** 2024-06-20

**Authors:** Nicolás Duque Clavijo, Juana Catalina Figueroa Aguirre, Claudia del Pilar Agudelo Lopez, Andrés Armando Borda, Beatriz Wills, Guillermo Enrique Quintero Vega

**Affiliations:** 1 Universidad de Los Andes, Hospital Universitario Fundación Santa Fe de Bogotá, Carrera 7 no.117– 15 Bogotá DC, Bogotá 110111, Colombia; 2 Hospital Universitario Fundación Santa Fe de Bogotá, Carrera 7 no.117– 15 Bogotá DC, Bogotá 110111, Colombia; 3 Universidad de los Andes, Hospital Universitario Fundación Santa Fe de Bogotá, Servicios Médicos de Hematología y Cardiología SAS, Bogotá 110111, Colombia; a https://orcid.org/0009-0009-4553-5168; b https://orcid.org/0000-0002-6837-3722

**Keywords:** diffuse large B cell lymphoma, hispanic, overall survival, prognosis, relapse

## Abstract

**Introduction:**

Diffuse large B-cell lymphoma (DLBCL), a prevalent non-Hodgkin lymphoma subtype, displays diverse clinical outcomes with persistently high mortality and relapse rates, despite treatment advancements. Notably, the Hispanic demographic lacks consideration in existing prognostic indices for DLBCL.

**Methods:**

A retrospective cohort study encompassing 112 DLBCL patients diagnosed between 2010 and 2020 was conducted at our institution. Patient data, including overall survival (OS), treatment response, and relapse, were analysed.

**Results:**

With a median age of 65 years and a predominant male population (60.7%), both the International Prognostic Index (IPI) and revised IPI correlated with OS. In multivariate analysis, patients with ki-67 ≥ 60% exhibited higher mortality risk (Hazard Ratio: 2.35, 95% confidence intervals (CI) 1.05–5.27, *p* = 0.039), even when controlled by IPI category and B2-microglobulin levels. The absence of B symptoms served as a protective factor for relapse (*p* < 0.01, OR: 0.147, 95% CI 0.058–0.376) when controlling for ki-67, CD5, and IPI.

**Conclusion:**

Our cohort demonstrated a 5-year OS rate comparable to high-income countries, highlighting the need for tailored prognostic models for Hispanic DLBCL patients. This study identifies easily accessible parameters aligning with regional resource constraints, providing insights into additional prognostic factors for DLBCL in the Hispanic population.

## Introduction

Diffuse large B-cell lymphoma (DLBCL) accounts for 30%–40% of all newly diagnosed cases worldwide and is the most prevalent subtype of non-Hodgkin lymphoma (NHL) [1]. The incidence of this condition is approximately 6 per 100,000 individuals in the US [2]. While the precise incidence rate in our region remains unknown, it is recognised that it represents a significant portion, comprising 40%–62% of all new NHL cases [3]. Meanwhile, the estimated prevalence in the United States spans between 63,000 and 143,000 [4]. Typically, it presents aggressively and progresses quickly. In most cases, patients with DLBCL manifest an enlarging mass in the neck, abdomen, or mediastinum, with nearly 30% presenting with B symptoms [5, 6]. In terms of the prognosis of these patients, the overall 5-year relative survival rate corresponds to approximately 62%–65% of the cases in the United States, which can change according to the risk [7, 8]. Furthermore, the disease exhibits a high degree of responsiveness to treatment, with a 60%–70% remission rate achieved and maintained among patients receiving Rituximab-based regimens. However, it should be noted that approximately 30%–40% of patients may experience relapse or refractory disease following first-line treatment [9, 10].

The two most commonly used prognostic indices are the International Prognostic Index (IPI) and its revised version, the revised IPI (R-IPI). The IPI was initially introduced in 1993 and had a broad range of outcomes. However, with the advent of Rituximab, there was a noticeable increase in survival rates, leading to the development of the R-IPI in 2007, which re-categorised patients into three risk groups instead of four [11]. Although both indices use the same variables, it has been 29 and 16 years since the IPI and R-IPI introduction, respectively. Yet, the development of these systems did not encompass the Hispanic population. Several authors have advocated for integrating novel variables identified within our patient population to potentially enhance the prognostic accuracy for Hispanic patients diagnosed with DLBCL [12, 13].

We conducted a retrospective cohort study in our institution with 112 patients diagnosed with DLBCL between 2010 and 2020. Our primary objective was to look for alternative clinical, laboratory, and demographic variables that statistically correlated with the primary clinical endpoint for our population, overall survival (OS). In addition, we also studied these parameters and their association with critical secondary endpoints, which included treatment response and relapsed disease. Besides the variables included in the IPI score, other variables have been described in medical literature and have shown a statistically significant association with OS in bivariate analysis. These variables include the gene expression profile, surface markers such as CD10 and CD38, MUM1, BCL-2, and BCL-6 gene expression, B2-microglobulin levels, the Ki-67, and the presence of B-symptoms, amongst others. Herein, we present the results from a retrospective cohort of 112 patients diagnosed with DLBCL and treated with Rituximab-based regimens.

## Methods

### Study design

We designed a retrospective, single center, cohort study in which we collected patient-level data on history of current illness, physical examination, pathology report, imaging, and laboratory parameters from our Institutional digital medical record. The Ethics Committee of the institution approved the study protocol. We collected imaging data, such as the disease stage and extranodal site involvement, directly from the reports. Similarly, we determined the immunohistochemistry markers and cellular proliferation index of the biopsies based solely on the information provided in the pathology reports, without any additional interpretation from us. We used Research Electronic Data Capture software for data collection and created a numerically coded database.

### Eligibility criteria

Inclusion criteria comprised patients 18 years or older with a histopathological diagnosis of DLBCL between 2010 and 2020 with access to a comprehensive medical record from our institution. We also included patients with DLBCL who had a strict follow-up, defined as having control for at least the previous 2 years in the case of living patients.

Exclusion criteria consisted of patients who started first-line treatment at another institution or those for whom we did not have full access to laboratory results at the time of the diagnosis. In addition, we excluded patients currently receiving treatment for a different type of cancer, those who started first-line therapy at our institution but continued it at another center, and those with primary central nervous system lymphoma.

### Assessments

The primary clinical endpoint was OS, defined as the time interval from the start of induction therapy to death from any cause or, in the case of living patients, until the date of their last check-up.

Positron emission tomography - Computed Tomography (PET-CT) and CT-Based response to therapy was determined according to the Lugano Revised Criteria for Response Assessment [14]. In addition, we defined relapse as a positive biopsy following a previously confirmed complete response documented through imaging studies.

### Statistical analysis

Statistical analysis was performed using the IBM SPSS statistics program, version 29.0. We used the Kaplan-Meier method to estimate OS in our study cohort [15]. Initially, we conducted bivariate analyses using either the chi-square or Fisher tests to determine statistical significance, based on sample size and data characteristics. These analyses were performed to identify associations between the variables examined and our primary and secondary clinical endpoints [16, 17]. Then, operating with statistically associated parameters in the bivariate analysis, we designed stratified Cox proportional hazard models, always using IPI as one of the stratification factors, to obtain adjusted hazard ratios with 95% confidence intervals (CIs) for OS [18]. To identify parameters associated with relapse, we first conducted a bivariate analysis using the chi-square test, followed by a multivariate analysis via a logistic regression [19].

## Results

### Demographic and baseline characteristics

This study analysed the records of 166 patients. Of these, 112 patients met the inclusion criteria and were included in the analysis. The median age of patients at diagnosis was 65 years, ranging from 24 to 95 years, with males comprising 60.7% of the patient population (Table 1). The majority of patients had Ann Arbor stage ≥3 (69.6%) and Eastern Cooperative Oncology Group (ECOG) performance status ≤1 (54.5%). Extranodal involvement was present in 78.5% of patients, with 33.9% having more than one site and 44.6% having only one site. The bone marrow was the most commonly involved extranodal site, followed by the gastrointestinal tract, accounting for 37.5% and 27.3% of the affected sites, respectively. As for lactate dehydrogenase (LDH), it was elevated in 43.8% of cases. Concerning IPI, patients were evenly distributed across all IPI risk categories, with 40.2% in the low and low intermediate risk categories, 46.4% in the high intermediate and high-risk categories, and 13.4% with unknown IPI risk group. On the other hand, with respect to R-IPI, only 3.6% of patients were categorised as being in the very good risk group whilst 36.6% and 46.4% pertained to the good and poor risk groups, respectively. Likewise, 13.4% of patients had an unknown R-IPI risk group.

Regarding the first-line treatment, the vast majority of patients received chemoimmunotherapy (97.3%), either as the sole treatment (88.2%) or in combination with either radiotherapy or surgery (8.4%). R-CHOP was the most commonly used regimen, with a frequency of 67.9%, followed by other rituximab-containing regimens, which accounted for 29.4% of the patients.

### OS, treatment response, and relapsed disease

**Figure 1.**
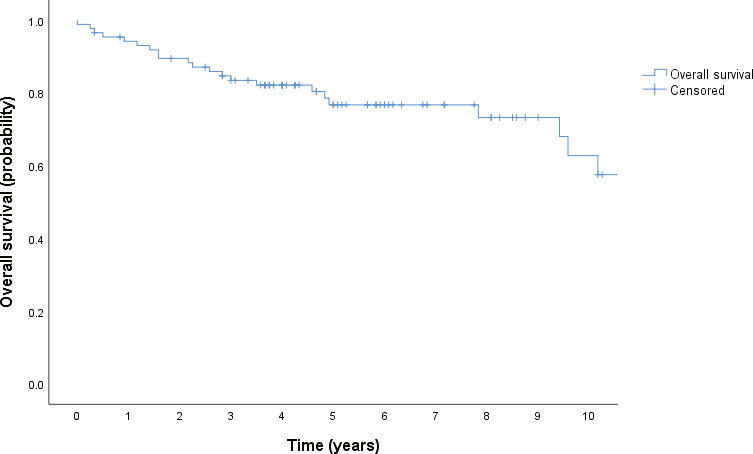
OS in years (Kaplan Meier).

**Figure 2.**
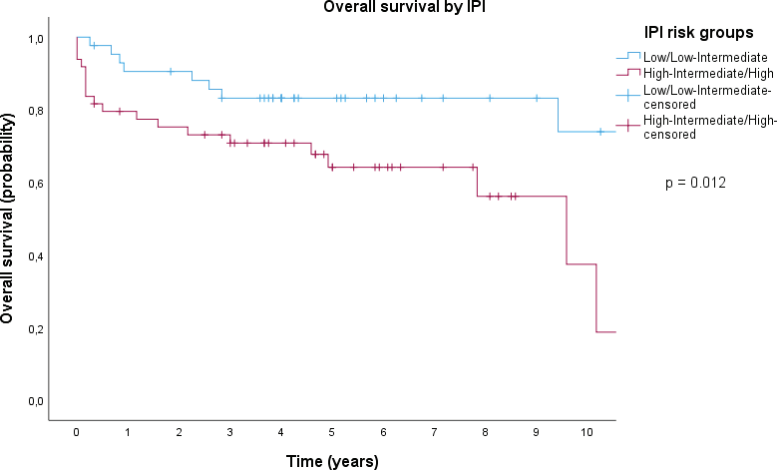
OS by IPI category (Kaplan Meier).

With a median follow-up of 4.25 years, there have been 35 deaths, and the median OS has not been reached; the 5-year OS estimate for the population was 68% (95% CI, 59–78), as shown in Figure 1. Patients in the low and low-intermediate risk categories were found to have higher estimated OS when compared to those in the high-intermediate and high-risk categories, as shown in Figure 2.

Regarding the first-line treatment response, 80.3% of patients had an overall response, with 70.5% responding entirely and 9.8% responding partially. On the other hand, 6.3% of patients progressed after first-line therapy, and 7.1% died during treatment. The remaining patients (6.3%) had an unknown first-line response status at the end of the follow-up. Furthermore, 33 patients (29.5%) subsequently received second-line treatment. Of these patients, the majority corresponded to patients who had relapsed (63.6%), followed by those who progressed (18.2%) or who partially responded to the first line (12.1%), and finally by patients who did not to receive a chemotherapy regimen as first-line therapy (6.1%). As for relapsed disease, out of 79 patients who responded completely to the first-line therapy, 29.1% relapsed, with a median duration between remission and relapse of 13 months. Infection was the leading cause of death (37.1%), followed by progression (17.1%) and cardiovascular causes (8.6%).

### Prognostic factors for OS

#### Bivariate analysis

The IPI score was validated using the cross-tabulation chi-square test for independence, reporting to be statistically significant (*p* < 0.001). Moreover, LDH showed a strong correlation with OS (*p* = 0.011). Most importantly, the presence of B-symptoms (*p* = 0.029) and elevated levels of B2-microglobulin (*p* = 0.04) were found to have a statistically significant negative impact on OS, according to a direct comparison using the chi-square method. While definitive statistical significance was not achieved, it is noteworthy that the presence of the surface marker CD10 and concurrent expression of BCL2 and BCL6 (double expressors) demonstrated a trend towards negatively impacting OS in a bivariate analysis, with *p*-values of 0.068 and 0.099, respectively.

#### Multivariate analysis

**Figure 3.**
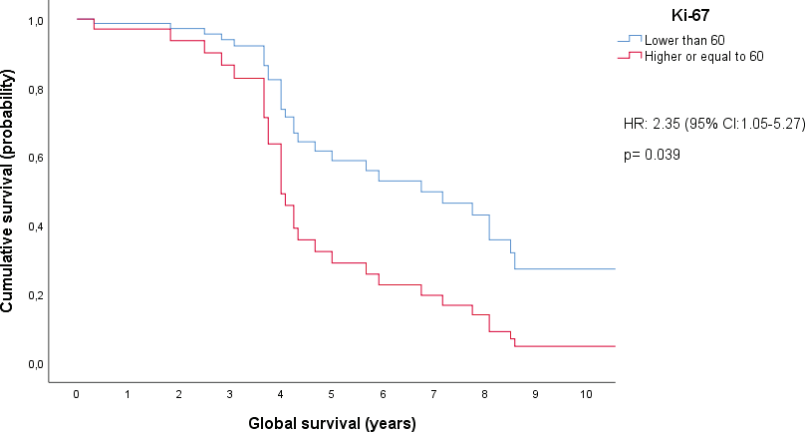
Cox proportional hazard model: ki-67 levels controlled by IPI categories and B2-microglobulin levels.

Variables that were statistically significant or marginally significant in the bivariate analysis were used to set up Cox proportional hazard models. When stratified by IPI category and B2-microglobulin levels, we found a statistically significant difference in OS between patients with a ki-67 proliferation index above or below 60%. This ki-67 cutoff has been previously reported as a discriminating prognosis for patients with DLBCL [20]. Notably, this analysis was conducted on a cohort of 44 patients. In fact, patients with ki-67 ≥ 60% had a higher chance of dying, with a hazard ratio of 2.35 (95% CI, 1.05–5.27) and a *p*-value of 0.039. The difference in OS between patients with ki-67 levels of 60% or greater and those with ki-67 levels below 60% is increased especially after 4 years from diagnosis. This difference is expected to continue growing larger until approximately 8.5 years after diagnosis, at which point it appears to stabilize, as shown in Figure 3.

### Prognostic factors for relapse

In terms of prognostic factors for relapse, we found a statistically significant association between several variables and higher relapse risk, as determined by bivariate analysis. These factors included the presence of B-symptoms (*p* = 0.014), surface marker CD5 (*p* = 0.021), high-IPI risk category (*p* = 0.025), and ki-67 ≥ 70 (*p* = 0.049). The multivariate analysis using logistic regression revealed that the absence of B symptoms functioned as a protective factor for relapse when controlling for ki-67, CD5, and the IPI, with a *p*-value <0.01 and an OR of 0.147 (95% CI, 0.058–0.376).

## Discussion

We reviewed the clinical characteristics, therapy approaches, survival, and relapse outcomes of DLBCL patients treated at our institution over the 2010–2020 decade. The cohort had a median age at diagnosis of 65 years, with a slight male predominance, similar to current literature on the subject [21]. Interestingly, we found a nearly equal distribution across IPI categories, despite a notable proportion showing an Ann Arbor stage of at least 3. Chemoimmunotherapy was the most commonly used approach, with R-CHOP being the most frequently used first-line treatment regimen, representing 67.9% of the cases. Altogether, rituximab-based regimens added up to 97.3% of our first-line therapy.

The cohort’s 5-year OS rate estimation was comparable to that of patients treated with standard chemoimmunotherapy in high-income countries [7, 22]. Indeed, 68% of patients were estimated to be alive 5 years after diagnosis; this survival was moderately higher than reported in other cohorts in our country (62.1%) [23]. As for prognostic factors, we validated the IPI, which was strongly associated with survival (*p* < 0.001). We also estimated OS over time by IPI categories, where we found a particularly significant difference between the low and low-intermediate risk groups compared to the high and high-intermediate risk groups. As a matter of fact, the 10-year survival rate of the lower risk groups was estimated to be more than three times that of the higher risk groups, as shown in Figure 2. Taken together, these findings provided compelling evidence that the original IPI retained its crucial role as a prognostic index in our patient population, underscoring its relevance and value in our region.

In the bivariate analysis, we encountered additional prognostic factors other than those established for the IPI. Specifically, B-symptoms and high levels of B2-microglobulin were found to potentially serve as additional unfavourable prognostic markers in patients with DLBCL. Moreover, the multivariate analysis indicated that patients with Ki-67 levels of 60% or higher had a significantly lower chance of survival compared to those with Ki-67 levels below 60%, when stratified by IPI category and B2-microglobulin levels. Indeed, after the fourth year following diagnosis, the estimated survival rate for the high Ki-67 group was approximately half of the survival rate estimated in the group with low Ki-67 levels. These results suggested that B2-microglobulin might be an important prognostic factor for OS and also pointed to a role and potential cut-off point for Ki-67 in patients with DLBCL. While previous studies successfully identified Ki-67’s impact on OS, this effect was solely evident in bivariate analysis, not in multivariate analysis [24–26]. Moreover, in contrast with our findings, previous studies have suggested higher cutoff points for defining a high Ki-67 expression level [25, 26]. Regarding B2-microglobulin, in a prior retrospective cohort study conducted in Japan, elevated levels were confirmed as a poor prognostic determinant for OS through both bivariate and multivariate analyses. Notably, this validation was conducted within a distinct patient demographic, and the prognostic model proposed in this study did not integrate the assessment of Ki-67 [27]. Further research involving a larger sample size is imperative to validate the statistical significance of surface marker CD10 and the concurrent expression of BCL2 and BCL6, as identified through immunohistochemistry, with regard to their correlation with OS.

Relapse was a common occurrence in DLBCL patients [28]. After performing a bivariate analysis to identify variables associated with relapse, we found several factors that were significantly associated with it, with the presence of B-symptoms at diagnosis being the strongest factor. This association remained significant after controlling for Ki-67, CD5, and IPI. Indeed, patients who presented with B-symptoms at the time of diagnosis were found to have a six-fold higher risk of relapse compared to those without, when controlling for these factors. Likewise, in a retrospective cohort study conducted in China with a sample size of 71 patients, there was a demonstrated statistically significant correlation between the presence of B-symptoms at diagnosis and subsequent relapse, evident in both bivariate and multivariate analyses [29]. While the correlation between CD5+ and unfavourable OS outcomes had been previously identified [30, 31], its association with relapse had not been previously acknowledged or integrated into a comprehensive multivariate model for assessing relapse. Concerning Ki-67 expression, although a statistically significant association with relapse had been previously established [26], our study evidenced its successful integration within a multivariate predictive model for relapse assessment. Consequently, investigating these factors as possible indicators for relapse might be advantageous for constructing a specific prognostic model for relapse.

Based on our study results, it was evident that a rituximab-based regimen can result in satisfactory responses in Hispanic patients with DLBCL. However, our study also suggested the importance of improving risk stratification, especially for those patients who might require innovative therapies or a more aggressive treatment regimen to improve their outcomes. Also, by identifying patients with factors associated with a higher risk of relapse, we could potentially more accurately suggest consolidation therapies and/or more strict follow-up visits, thereby possibly improving their OS.

## Conclusion

Elevated levels of ki-67, of 60% or higher, might be an important marker for predicting poor survival outcomes in DLBCL, when controlling for IPI category and B2-microglobulin levels. For relapse, the presence of B-symptoms at the time of diagnosis may serve as an indicator for predicting an increased risk, when controlling for ki-67, CD5, and IPI. This investigation highlighted additional prognostic factors influencing survival and relapse among Hispanic individuals diagnosed with DLBCL, an understudied demographic within this condition. A promising aspect of this study is its proposition of novel, readily accessible parameters, that aligning with the resource-constrained context prevalent in our region.

## Suplementary materials

For original data, please contact n.duquec@uniandes.edu.co

## Conflicts of interest

The authors declare that they have no known competing financial interests or personal relationships, including grants, fellowships, commercial assistance, or financial sponsorship, that could have appeared to influence the work reported in this paper.

## Funding

This research did not receive any specific grant from funding agencies in the public, commercial, or not-for-profit sectors.

## Research involving human participants and ethics approval

The study was performed in line with the principles of the Declaration of Helsinki. Approval was granted by the Ethics Committee of the Fundación Santa Fe de Bogotá (CCEI 15583-2023).

## Author contributions

NDC designed the study, collected, analysed and interpreted the data, performed statistical analyses, and wrote the manuscript; JCFA designed the study, collected, analysed, interpreted the data, and wrote the manuscript; CPAL reviewed drafts of the manuscript and approved the final version; AAB reviewed drafts of the manuscript and approved the final version; BW edited and reviewed the manuscript, and provided final approval of the study; GEQV conceived the study, edited and reviewed the manuscript, and provided final approval of the study.

## Figures and Tables

**Table 1.** Baseline patient characteristics.

Characteristics	Total (*n* = 112)
Median age (range)	65 (24–95)
Sex	
Male	68 (60.7%)
Female	44 (39.3%)
ECOG	
0–1	61 (54.5%)
2–4	46 (41.1%)
Missing	5 (4.5%)
Disease stage	
I–II	27 (24.1%)
III–IV	78 (69.6%)
Missing	7 (6.3%)
Extranodal sites	
0	21 (18.8%)
1	50 (44.6%)
>1	38 (33.9%)
Missing	3 (2.7%)
LDH ratio	
0–1 × ULN	46 (41.1%)
>1 × ULN	49 (43.8%)
Missing	17 (15.2%)
IPI risk group	
Low risk	25 (22.3%)
Low-intermediate risk	20 (17.9%)
High- intermediate risk	25 (22.3%)
High risk	27 (24.1%)
Missing	15 (13.4%)
R-IPI risk group	
Very good	4 (3.6%)
Good	41 (36.6%)
Poor	52 (46.4%)
Missing	15 (13.4%)
B symptoms	
No	70 (62.5%)
Yes	38 (33.9%)
Missing	4 (3.6%)
Phenotype	
Germinal center	56 (50.0%)
Activated	29 (25.9%)
Other	11 (9.8%)
Missing	16 (14.3%)
Expression of BCL2/BCL6 on IHC*	
Not expressor or single expressor	40 (35.7%)
Double expressor	50 (44.6%)
Missing	22 (19.6%)
Ki-67	
<60%	24 (21.4%)
≥60	77 (68.8%)
Missing	11 (9.8%)
Beta-2 microglobulin ratio	
0–1 × ULN	27 (24.1%)
>1 ULN	23 (20.5%)
Missing	62 (55.4%)
First line regimen	
R-CHOP	76 (67.9%)
Other rituximab containing regimens †	33 (29.4%)
Did not receive chemotherapy	3 (2.7%)
*Immunohistochemistry, ^†^Including R-CHOEP, R-DA-CHOEP, R-CVP, R-Bendamustine and R-CNOP	
